# Antimicrobial Susceptibility of Autochthonous Aquatic *Vibrio cholerae* in Haiti

**DOI:** 10.3389/fmicb.2016.01671

**Published:** 2016-10-21

**Authors:** Sandrine Baron, Jean Lesne, Eric Jouy, Emeline Larvor, Isabelle Kempf, Jacques Boncy, Stanilas Rebaudet, Renaud Piarroux

**Affiliations:** ^1^Mycoplasmology-Bacteriology Unit, Ploufragan-Plouzané Laboratory, French Agency for Food, Environmental and Occupational Health & SafetyPloufragan, France; ^2^Vie-Agro-Santé, Bretagne-Loire UniversityRennes, France; ^3^National Public Health Laboratory, Ministry of Public Health and PopulationPort au Prince, Haiti; ^4^IT-TPT UMR MD3, Aix-Marseille UniversityMarseille, France

**Keywords:** *Vibrio cholerae* non-O1/non-O139, antimicrobial resistance, Haiti, aquatic environment, cholera

## Abstract

We investigated the antimicrobial susceptibility of 50 environmental isolates of *Vibrio cholerae* non-O1/non-O139 collected in surface waters in Haiti in July 2012, during an active cholera outbreak. A panel of 16 antibiotics was tested on the isolates using the disk diffusion method and PCR detection of seven resistance-associated genes (*strA*/*B*, s*ul1*/*2, ermA*/*B*, and *mefA*). All isolates were susceptible to amoxicillin-clavulanic acid, cefotaxime, imipenem, ciprofloxacin, norfloxacin, amikacin, and gentamicin. Nearly a quarter (22.0%) of the isolates were susceptible to all 16 antimicrobials tested and only 8.0% of the isolates (*n* = 4) were multidrug-resistant. The highest proportions of resistant isolates were observed for sulfonamide (70.0%), amoxicillin (12.0%), and trimethoprim-sulfamethoxazole (10.0%). One strain was resistant to erythromycin and one to doxycycline, two antibiotics used to treat cholera in Haiti. Among the 50 isolates, 78% possessed at least two resistance-associated genes, and the genes *sul1, erm*A, and *str*B were detected in all four multidrug-resistant isolates. Our results clearly indicate that the autochthonous population of *V. cholerae* non-O1/non-O139 found in surface waters in Haiti shows antimicrobial patterns different from that of the outbreak strain. The presence in the Haitian aquatic environment of *V. cholerae* non-O1/non-O139 with reduced susceptibility or resistance to antibiotics used in human medicine may constitute a mild public health threat.

## Introduction

Reports on clinical strains of *Vibrio cholerae* O1 resistant to commonly used antibiotics are on the rise (Garg et al., 2000, 2001; Ghosh and Ramamurthy, 2011; Harris et al., 2012). Antibiotic resistance is a global health concern because resulting infections can be more difficult to treat. The increase in resistance to antimicrobial drugs can result either from the accumulation of genetic mutations, following exposure of the circulating bacteria to antibiotics during the medical treatment of epidemics, or from the acquisition of resistance genes, through the mobilization and exchange of a variety of genetic elements. In the case of *V. cholerae*, self-transmissible mobile genetic elements may harbor an SXT constin (a large conjugative element), which may confer resistance to sulfamethoxazole, trimethoprim, chloramphenicol, and streptomycin (Hochhut et al., 2001).

Surprisingly, data on the susceptibility of environmental isolates of *V. cholerae* non-O1/non-O139 are still scarce (Kumar et al., 2009; Bier et al., 2015; Bhuyan et al., 2016). However, knowledge on the prevalence of antimicrobial resistance in these serogroups is of global health interest for two reasons.

First, contrary to O1/O139, non-O1/non-O139 serogroups are detected worldwide in all types of waters: freshwater, estuarine, saline water, and wastewater. Aquatic environments may provide an ideal setting for the acquisition and dissemination of antibiotic resistance: (i) they are frequently affected by anthropogenic activities (Marti et al., 2014); (ii) they contain autochthonous bacterial flora that may harbor resistance-associated genes; (iii) they bring bacteria from different origins (human, livestock, etc.) into contact with each other; (iv) they can contain antimicrobials or biocides which may select for resistant bacteria. Wastewater constitutes a hot spot for the emergence of antimicrobial resistance (Rizzo et al., 2013), particularly for *V. cholerae*, which has a dual life cycle (intestinal and aquatic); (Schoolnik and Yildiz, 2000). Therefore, due to its specific genetic abilities, and its ecological characteristics, *V. cholerae* may be an important vehicle of transmission of resistance genes in all aquatic environments either within bacterial species or between bacterial genera. In countries with endemic cholera, these autochthonous aquatic serogroups of *V. cholerae* can also co-infect cholera cases, providing an opportunity for the exchange of antimicrobial resistance genes with clinical strains of serogroup O1 in the human intestinal lumen. Furthermore, the intestinal epidemic clones circulating in the environment can theoretically exchange resistance genes with the autochthonous populations of *V. cholerae*.

Second, some clones of *V. cholerae* non-O1/non-O139 from aquatic environments may be opportunist pathogens, which can cause gastro-intestinal or extra-intestinal infections (bacteremia, septicemic, otitis, etc.) (Lukinmaa et al., 2006; Deshayes et al., 2015). Throughout the world, the number of cases of *V. cholerae* non-O1/non-O139 infections are rising (Baker-Austin et al., 2013); moreover, reports of very severe infections are becoming more frequent, with even one death recently reported in Austria (Hirk et al., 2016). In Haiti, *V. cholerae* non-O1/non-O139 has been shown to be a local intestinal infectious pathogen. Very early in the on-going Haitian cholera outbreak (November 2010), a microbiological investigation of 81 clinical cases of acute diarrhea with varying severity from 18 towns showed that *V. cholerae* non-O1/non-O139 was isolated from 28% of the stool specimens, either alone (*n* = 17) or in co-culture with toxigenic *V. cholerae* O1 (*n* = 5). The latter cases were confirmed cholera cases, as were 39 other cases with the presence of toxigenic *V. cholerae* O1 alone (Hasan et al., 2012). However two years later (from April 2012 to March 2013), systematic testing of profuse watery diarrhea cases was carried out in four hospitals that had cholera treatment facilities. Among the 1616 specimens of stools tested in culture, 60% were positive for toxigenic *V. cholerae* O1, one gave a non-toxigenic isolate of *V. cholerae* O1, but none were positive for *V. cholerae* non-O1/non-O139 (Steenland et al., 2013). Other studies on stools collected from patients during the Haiti outbreak found only serogroup O1 isolates (Talkington et al., 2011; Katz et al., 2013). These results are not contradictory: non-O1/non-O139 serogroups can cause a diarrheal disease that is generally less severe than cholera and do not particularly have epidemic potential (Menon et al., 2009). Nevertheless, the pathogenic clones of *V. cholerae*, whether they are agents of cholera or of milder infections, can circulate via aquatic environments and some authors even speculate that toxigenic clones of *V. cholerae* are autochthonous to estuaries and rivers systems worldwide as are other clones (Colwell, 1996).

We therefore investigated the antimicrobial susceptibility of 50 environmental isolates of non-toxigenic *V. cholerae* collected in various surface waters in Haiti in July 2012, during the active cholera epidemic (Baron et al., 2013). The aim of this study was to document, in this context, the susceptibility of strains belonging to non-O1/non-O139 serogroups to 16 antibiotics used and compare it to those of the Haitian epidemic strain.

## Materials and methods

### Collection of strains

In a previous study, water samples, including wastewater, were collected from 35 stations described in Baron et al. (2013). *V. cholerae* was detected by culturing samples on TCBS (thiosulfate citrate bile sucrose) agar (Difco, BD Biosciences, Le pont de Claix, France) after enrichment in peptone alkaline saline water (41°C ± 1 for 16–24 h; Muic, 1990). Presumptive identification of *V. cholerae* was given to all sucrose-fermenting isolates that were able to grow on nutrient agar without added NaCl, and that tested positive for oxidase (Baron et al., 2007). Presumptive *V. cholerae* were isolated from 27 of the 35 stations sampled, but isolates from six stations could not be regrown (Table 1).

**Table 1:** **Distribution of the 50 isolates of confirmed *V. cholerae* non-O1/non-O139 and characteristics of the 27 stations (see Baron et al., 2013 for correspondence)**.

**Sampling stations**				**Water characteristics**			**Number of isolates**
**Department**	**Town**	**Location**	**ID^a^**	**Salinity (%0)^b^**	**Fecal contamination:**		
					***E. coli* /100 mL**	**Group^d^**	
West (Metropolitan area)	Port-au-Prince	Martissant (street wastewater)	2	ND^c^	ND	HFC	2
West (Metropolitan area)	Carrefour	Mariani (wastewater in the river)	32	0.21	106,000	HFC	NG^e^
West (Metropolitan area)	Carrefour	Mariani (river shore)	33	0.20	35,000	HFC	2
West (Metropolitan area)	Carrefour	Mariani (river shore)	34	0.21	46,000	HFC	3
West (Metropolitan area)	Carrefour	Mariani (macrophyte lagoon)	35	25.02	12,400	HFC	NG
West (Metropolitan area)	Carrefour	Mariani (macrophyte lagoon)	36	10.72	50,000	HFC	NG
Artibonite	Gonaives	Small canal of wastewater	19	2.09	91,000	HFC	4
Artibonite	Gonaives	Large canal of wastewater	24	1.40	36,000	HFC	3
West	Thomazeau	Trou Caïman Lake	3	1.27	ND	LFC	3
West	Thomazeau	Etang Saumâtre Lake (shore)	4	4.90	ND	LFC	3
West	Thomazeau	Etang Saumâtre Lake (far from the shore)	5	5.62	ND	LFC	2
Artibonite	Saint Marc	Etang Bois-Neuf	18	12.41	2000	LFC	5
Artibonite	Saint Marc	Pont-Sondé (Artibonite River)	6	0.14	4800	LFC	4
Artibonite	Grande Saline	Main canal 1	17	0.15	2800	LFC	2
Artibonite	Grande Saline	Main canal 2	16	0.15	3600	LFC	2
Artibonite	Grande Saline	Drouin—main canal 3 (point-of-use)	7	0.15	2100	LFC	2
Artibonite	Grande Saline	Artibonite River estuary 1	9	0.14	2100	LFC	1
Artibonite	Grande Saline	Artibonite River estuary 2	10	0.15	3100	LFC	1
Artibonite	Grande Saline	Basin 1	14	0.75	1000	LFC	1
Artibonite	Grande Saline	Basin 2	15	0.27	< 100	LFC	4
Artibonite	L'Estère	L'Estère (river)	25	0.15	300	LFC	1
Artibonite	L'Estère	L'Estère (small canal)	26	0.15	400	LFC	1
Artibonite	L'Estère	L'Estère (large canal)	27	0.14	300	LFC	1
Artibonite	L'Estère	L'Estère (roadside)	28	0.37	4700	LFC	NG
Artibonite	Desdunes	Route de Desdunes (small canal)	29	0.26	200	LFC	NG
Artibonite	Desdunes	Route de Desdunes (large canal)	30	0.52	300	LFC	3
Artibonite	Desdunes	Route de Desdunes (rice field)	31	0.30	< 100	LFC	NG

a ID, identification number of the station;

b salinity: fresh water < 0.5%¸; brackish water 0.5–16%¸;

c ND, no data;

d HFC, high fecal contamination: E. coli >10^4^ CFU/100 mL; LFC, low fecal contamination: E. coli ≤ 10^4^ CFU/100 mL;

e NG, isolate from the National Health of Public Health in Haiti did not grow in the ANSES laboratory.

Fecal contamination (FC) was determined using Petrifilm™ Select *Escherichia coli* (Département Microbiologie Laboratoires 3M Santé, Cergy, France). Based on the FC level (Table 1), the 21 sampled stations were divided into two groups. The high FC (HFC) group for which the density of *E. coli* was at least 10^4^ CFU/100 mL included five stations that were all wastewaters. The low FC (LFC) group, for which the *E. coli* density level was < 10^4^ CFU/100 mL, included Trou Caïman Lake (Station 3) and Etang Saumâtre Lake (Stations 4 and 5; Table 1). Conductivity was assessed at the laboratory with a field conductometer (Hanna HI-99301, Grosseron, Nantes, France).

### Confirmation and characterization of *V. cholerae*

Agglutination using a polyclonal antibody specific to the O1 surface antigen (Bio-Rad, Marnes-la-Coquette, France) was performed on presumptive *V. cholerae* isolates at the Haiti National Public Health Laboratory. A saline solution was used as a control to identify self-agglutinating isolates. One isolate per enrichment and per station was conserved and sent to the ANSES-Laboratory of Ploufragan-Plouzané for further analysis. The identification of presumptive *V. cholerae* was confirmed by PCR (Nandi et al., 2000). The genes coding for the O1 and O139 surface antigens (*rfb*) were assessed with PCR using O1- and O139-specific primers (Hoshino et al., 1998; Table 2). The cholera toxin gene c*txA* was screened using PCR (Hoshino et al., 1998; Nandi et al., 2000).

**Table 2:** **List of primers used in this study**.

**Targeted gene**	**Primer name**	**Primer sequence (5′–3′)**	**T°C^a^**	**Amplicon size (bp)**	**References**
*ermA*	ermA1	TAACATCAGTACGGATATTG	54	139	Di Cesare et al., 2013
	ermA2	AGTCTACACTTGGCTTAGG			
*ermB*	ermB1	CCGAACACTAGGGTTGCTC	54	200	Di Cesare et al., 2013
	ermB2	ATCTGGAACATCTGTGGTATG			
*mef*	mef1	AGTATCATTAATCACTAGTGC	54	348	Di Cesare et al., 2013
	mef2	TTCTTCTGGTACTAAAAGTGG			
*sul1*	Sul1-1	CGCACCGGAAACATCGCTGCAC	65	162	Pei et al., 2006
	Sul1-2	TGAAGTTCCGCCGCAAGGCTCG			
*sul2*	Sul2-1	TCCGGTGGAGGCCGGTATCTGG	57.5	190	Pei et al., 2006
	Sul2-2	CGGGAATGCCATCTGCCTTGAG			
O139 *rfb*	O139-1	AGCCTCTTTATTACGGGTGG	55	449	Hoshino et al., 1998
	O139-2	GTCAAACCCGATCGTAAAGG			
O1 *rfb*	O1-1	GTTTCACTGAACAGATGGG	55	192	Hoshino et al., 1998
	O1-2	GGTCATCTGTAAGTACAAC			
*ctxA*	ctx Ats	CTCAGACGGGATTTGTTAGGCACG	64	301	Nandi et al., 2000
	ctx A	TCTATCTCTGTAGCCCCTATTACG			
*ompW*	ompW ts	CACCAAGAAGGTGACTTTATTGTG	64	588	Nandi et al., 2000
	ompW ta	GAACTTATAACCACCCGCG			
*strA*	strA-F	GAGAGCGTGACCGCCTCATT	57	862	Popowska et al., 2012
	strA-R	TCTGCTTCATCTGGCGCTGC			
*strB*	strB-F	GCTCGGTCGTGAGAACAATC	54	859	Popowska et al., 2012
	strB-R	AGAATGCGTCCGCCATCTGT			

a Annealing temperature.

### Antibiotic resistance profiles

The susceptibility of *V. cholerae* isolates was tested using the disk diffusion method according to Clinical and Laboratory Standards Institute (CLSI) guidelines (CLSI, 2010a) for the 16 following antimicrobial agents: ampicillin, amoxicillin-clavulanic acid, cefotaxime, imipenem, chloramphenicol, nalidixic acid, ciprofloxacin, norfloxacin, amikacin, gentamicin, streptomycin, tetracycline, doxycycline, sulfonamide, trimethoprim-sulfamethoxazole, and erythromycin (Table 3). *E. coli* ATCC 25922 served as a positive control. The CLSI interpretative criteria for disk diffusion susceptibility testing of *Vibrio* spp. (CLSI, 2010a) were used when available. For nalidixic acid, norfloxacin, amikacin, and streptomycin, the interpretative criteria for *Enterobacteriaceae* were used (CLSI, 2016; Table 3). No criteria were available for erythromycin or doxycycline; therefore, the distribution of the inhibition diameters was recorded and interpretation was based on obtained distribution plots. The separation between wild-type (microorganisms without acquired resistance mechanisms) and non-wild-type populations (microorganisms with acquired resistance mechanisms) was determined by visual inspection of the diameter distribution (Hombach et al., 2014). Wild-type populations were considered as susceptible populations and non-wild-type as resistant populations. The few intermediate results were categorized as resistant for this study.

**Table 3:** **Interpretative criteria used to determine antimicrobial susceptibility with the disk diffusion test in *Vibrio cholerae* isolates**.

**Antimicrobial class**	**Antimicrobial agent**	**Abbreviation**	**Disk content (μg)**	**Zone diameter interpretative criteria (mm)**			**References**
				**Susceptible**	**Intermediate**	**Resistant**	
ß-lactams	Ampicillin	AM	10	≥17	14–16	≤ 13	CLSI, 2010a
	Amoxicillin-clavulanic acid	AMC	20/10	≥18	14–17	≤ 13	CLSI, 2010a
	Cefotaxime	CTX	30	≥26	23–25	≤ 22	CLSI, 2010a
	Imipenem	IPM	10	≥23	20–22	≤ 19	CLSI, 2010a
Phenicols	Chloramphenicol	C	30	≥18	13–17	≤ 12	CLSI, 2010a
Aminoglycosides	Amikacin	AMK	30	≥17	15–16	≤ 14	CLSI, 2010a
	Gentamicin	GEN	10	≥15	13–14	≤ 12	CLSI, 2010a
	Streptomycin	STR	10	≥17	13–16	≤ 12	CLSI, 2016
Quinolones	Ciprofloxacin	CIP	5	≥21	16–20	≤ 15	CLSI, 2010a
	Nalidixic acid	NA	30	≥19	14–18	≤ 13	CLSI, 2016
	Norfloxacin	NOR	10	≥17	13–16	≤ 12	CLSI, 2016
Folate pathway inhibitors	Sulfonamide	SSS	300	≥17	13–16	≤ 12	CLSI, 2010a
	Trimethoprim-sulfamethoxazole	SXT	1.25/23.75	≥16	11–15	≤ 10	CLSI, 2010a
Tetracyclines	Tetracycline	TET	30	≥15	12–14	≤ 11	CLSI, 2010a
	Doxycycline	DO	30	–	–	–	
Macrolides	Erythromycin	ERY	15	≥17	13–16	≤ 12	CLSI, 2016

Interpretative criteria specific for Vibrio spp., including V. cholerae described in CLSI document M45 3rd edition (CLSI, 2010a) are adapted from those for Enterobacteriaceae M100 25S (CLSI, 2010b). For streptomycin, norfloxacin and nalidixic acid breakpoints described for Enterobacteriaceae in M100 26S (CLSI, 2016) were used. No breakpoints were available for doxycycline or for erythromycin.

Genes associated with resistance to streptomycin (*strA* and *strB*) and to sulfonamide (*sul1* and *sul2*)-which may be associated with the presence of *V. cholerae* SXT-as well as genes associated with erythromycin resistance (*ermA, ermB*, and *mefA*)-an antimicrobial agent used in Haiti for cholera treatment-were detected using PCR (Pei et al., 2006; Popowska et al., 2012; Di Cesare et al., 2013). Class 1, 2, and 3 integrons were screened using PCR (Barraud et al., 2010). All primer pairs, target genes, corresponding annealing temperatures, and amplicon sizes are listed in Table 2.

## Results

### Collection of isolates

The collection was composed of 50 isolates of *V. cholerae* from 21 stations (Table 1): 36 (72.0%) were isolated from the 16 LFC stations and 14 from the 5 HFC stations (for description of stations see Baron et al., 2013). None of the 50 *V. cholerae* isolates belonged to O1 or O139 serogroups nor produced the cholera toxin (i.e., the *ctxA* gene was not detected).

### Antibiotic resistance phenotypes

All tested isolates were susceptible to seven antibiotics of the tested panel: amoxicillin/clavulanic acid, cefotaxime, imipenem, ciprofloxacin, norfloxacin, amikacin, and gentamicin. Eleven isolates (22.0%) were susceptible to all 16 tested antibiotics (three isolates out of 14 from the HFC group and eight out of 36 from the LFC group). The highest proportions of resistant isolates were observed for sulfonamide (70.0%), ampicillin (12.0%), and trimethoprim-sulfamethoxazole (10.0%; Table 4).

**Table 4:** **Phenotypic and genotypic profiles of susceptibility in the 50 strains of *V. cholerae* non-O1/non-O139**.

			**Phenotypic antimicrobial suceptibility^c^**										**Molecular determinants^d^**							
**Strain**	**Station**	**Group^a^**	**Resistance profile**	**SSS**	**STR**	**E^b^**	**DO^b^**	**TET**	**AM**	**NA**	**SXT**	**C**	***sul1***	***sul2***	***strA***	***strB***	***ermA***	***ermB***	***mefA***	**Integron**
VCH3	2	HFC	C−SXT−AM−TET−SSS−STR−E	R	R	7^*^	24	R	R	S	R	R	+	−	+	+	+	−	−	+
VCH126	19	HFC	C−SXT−TET−SSS−STR−DO	R	R	22	15^*^	R	S	S	R	R	+	+	+	+	+	−	−	−
VCH85	25	LFC	C−SXT−SSS−STR	R	R	18	25	S	S	S	R	R	+	+	+	+	+	−	−	−
VCH55	17	LFC	AM−SSS−STR	R	R	21	29	S	R	S	S	S	+	−	−	−	−	−	−	−
VCH23	6	LFC	AM−SSS	R	S	22	29	S	R	S	S	S	+	−	−	+	+	−	−	−
VCH28	7	LFC	AM−SSS	R	S	16	27	S	R	S	S	S	+	−	−	−	+	−	−	−
VCH59	18	LFC	AN−SSS	R	S	22	31	S	S	R	S	S	+	−	−	−	+	−	−	−
VCH12	3	LFC	SSS−STR	R	R	18	27	S	S	S	S	S	+	−	−	−	+	−	−	−
VCH113	33	HFC	SXT−SSS	R	S	18	27	S	S	S	R	S	+	−	−	−	+	−	−	−
VCH13	5	LFC	SSS	R	S	18	29	S	S	S	S	S	+	−	−	+	+	−	−	−
VCH66	18	LFC	SSS	R	S	21	26	S	S	S	S	S	+	−	−	−	+	−	−	−
VCH72	19	HFC	SSS	R	S	20	27	S	S	S	S	S	+	−	−	−	−	−	−	−
VCH4	4	LFC	SSS	R	S	20	27	S	S	S	S	S	+	−	−	−	+	−	−	−
VCH31	9	LFC	SSS	R	S	19	28	S	S	S	S	S	+	−	−	−	+	−	−	−
VCH107	30	LFC	SSS	R	S	18	27	S	S	S	S	S	−	−	−	+	+	−	−	−
VCH5	4	LFC	SSS	R	S	17	29	S	S	S	S	S	+	−	−	+	+	−	−	−
VCH30	7	LFC	SSS	R	S	23	28	S	S	S	S	S	+	−	−	−	−	−	−	−
VCH41	15	LFC	SSS	R	S	21	28	S	S	S	S	S	−	−	−	−	+	−	−	−
VCH45	15	LFC	SSS	R	S	18	29	S	S	S	S	S	+	−	−	−	+	−	−	−
VCH52	16	LFC	SSS	R	S	21	31	S	S	S	S	S	−	−	−	−	+	−	−	−
VCH57	17	LFC	SSS	R	S	22	28	S	S	S	S	S	+	−	−	−	−	−	−	−
VCH65	18	LFC	SSS	R	S	21	28	S	S	S	S	S	−	−	−	−	+	−	−	−
VCH76	24	HFC	SSS	R	S	20	29	S	S	S	S	S	+	−	−	−	+	−	−	−
VCH79	24	HFC	SSS	R	S	20	29	S	S	S	S	S	+	−	−	−	+	−	−	−
VCH81	24	HFC	SSS	R	S	23	29	S	S	S	S	S	+	−	−	−	+	−	−	−
VCH90	27	LFC	SSS	R	S	17	29	S	S	S	S	S	+	+	−	+	+	−	+	−
VCH104	30	LFC	SSS	R	S	21	29	S	S	S	S	S	−	−	−	+	−	−	−	−
VCH2	2	HFC	SSS	R	S	18	28	S	S	S	S	S	−	−	−	+	+	−	−	−
VCH9	3	LFC	SSS	R	S	22	29	S	S	S	S	S	+	−	−	−	+	−	−	−
VCH102	30	LFC	SSS	R	S	19	32	S	S	S	S	S	+	−	−	−	+	−	−	−
VCH112	33	HFC	SSS	R	S	21	29	S	S	S	S	S	+	−	−	−	+	−	−	−
VCH48	15	LFC	SSS	R	S	23	30	S	S	S	S	S	+	−	−	−	+	−	−	−
VCH50	16	LFC	SSS	R	S	22	31	S	S	S	S	S	+	−	−	−	+	−	−	−
VCH117	34	HFC	SSS	R	S	21	30	S	S	S	S	S	+	−	−	−	+	−	−	−
VCH146	4	LFC	SSS	R	S	23	30	S	S	S	S	S	+	−	−	−	+	−	−	−
VCH17	5	LFC	AM	S	S	24	30	S	R	S	S	S	+	−	−	+	+	−	−	−
VCH26	6	LFC	AM	S	S	21	31	S	R	S	S	S	+	−	−	−	+	−	−	−
VCH69	19	HFC	AN	S	S	22	29	S	S	R	S	S	+	−	−	−	+	−	−	−
VCH61	18	LFC	SXT	S	S	23	26	S	S	S	R	S	−	−	−	−	+	−	−	−
VCH22	6	LFC	S	S	S	20	32	S	S	S	S	S	+	−	−	+	+	−	−	−
VCH7	3	LFC	S	S	S	20	30	S	S	S	S	S	+	−	−	−	+	−	−	−
VCH34−1	10	LFC	S	S	S	19	32	S	S	S	S	S	−	−	−	+	+	−	−	−
VCH40	14	LFC	S	S	S	19	30	S	S	S	S	S	−	−	−	−	+	−	−	−
VCH44	15	LFC	S	S	S	20	30	S	S	S	S	S	+	−	−	−	+	−	−	−
VCH116	34	HFC	S	S	S	20	30	S	S	S	S	S	+	−	−	−	+	−	−	−
VCH62	18	LFC	S	S	S	22	32	S	S	S	S	S	+	−	−	−	+	−	−	−
VCH70	19	HFC	S	S	S	21	34	S	S	S	S	S	+	−	−	−	+	−	−	−
VCH89	26	LFC	S	S	S	14	30	S	S	S	S	S	−	−	−	−	+	−	−	−
VCH131	34	HFC	S	S	S	21	32	S	S	S	S	S	+	−	−	+	+	−	−	−
VCH20	6	LFC	S	S	S	17	31	S	S	S	S	S	+	−	−	−	+	−	−	−

SSS, sulfonamide; STR, streptomycin; E, erythromycin; DO, doxycycline; TET, tetracycline; AM, ampicillin; NA, nalidixic acid; SXT, trimethoprim-sulfamethoxazole and C, chloramphenicol; R, resistant; S, susceptible; all intermediate results were considered as resistant.

a Based on the fecal contamination level, the 21 sampled sites were divided into two groups. High fecal contamination (HFC) with E. coli >10^4^ CFU/100 mL; low fecal contamination (LFC) with E. coli ≤ 10^4^ CFU/100 mL.

b For doxycycline and erythromycin, as no interpretative criteria was available, the diameter of inhibition is given.^*^ indicates that the isolate was resistant to this antimicrobial agent.

c All the isolates were susceptible to amoxicillin/clavulanic acid, amikacin, gentamicin, cefotaxime, ciprofloxacin, imipenem and norfloxacin, and were not included in this table.

d +, gene/integron was detected by PCR; -, gene/integron was not detected by PCR.

**Figure 1:**
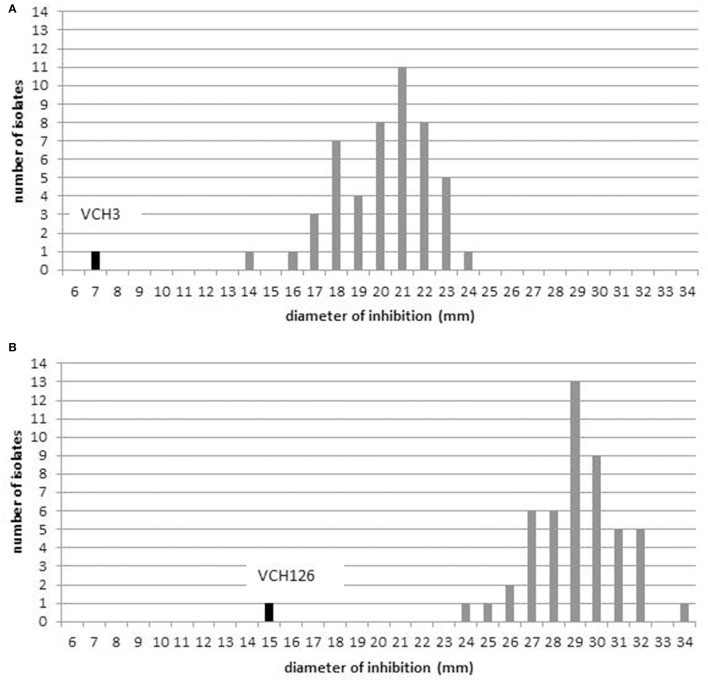
**Distribution of diffusion zone diameters: (A) erythromycin (15 μg); (B) doxycycline (30 μg)**.

One isolate, VCH126 from HFC station 19, showed a smaller inhibition zone (15 mm) for doxycycline than the 49 other isolates (Figure 1B) and was resistant to tetracycline. Given that the results from the tetracycline disk are typically used to predict susceptibility to doxycycline (Centers for Disease Control Prevention, 1999), we concluded that VCH126 was resistant to doxycycline. VCH3, isolated from LFC station 2, showed an inhibition zone of 7 mm for erythromycin (Figure 1A); this isolate was declared resistant to erythromycin.

**Figure 2:**
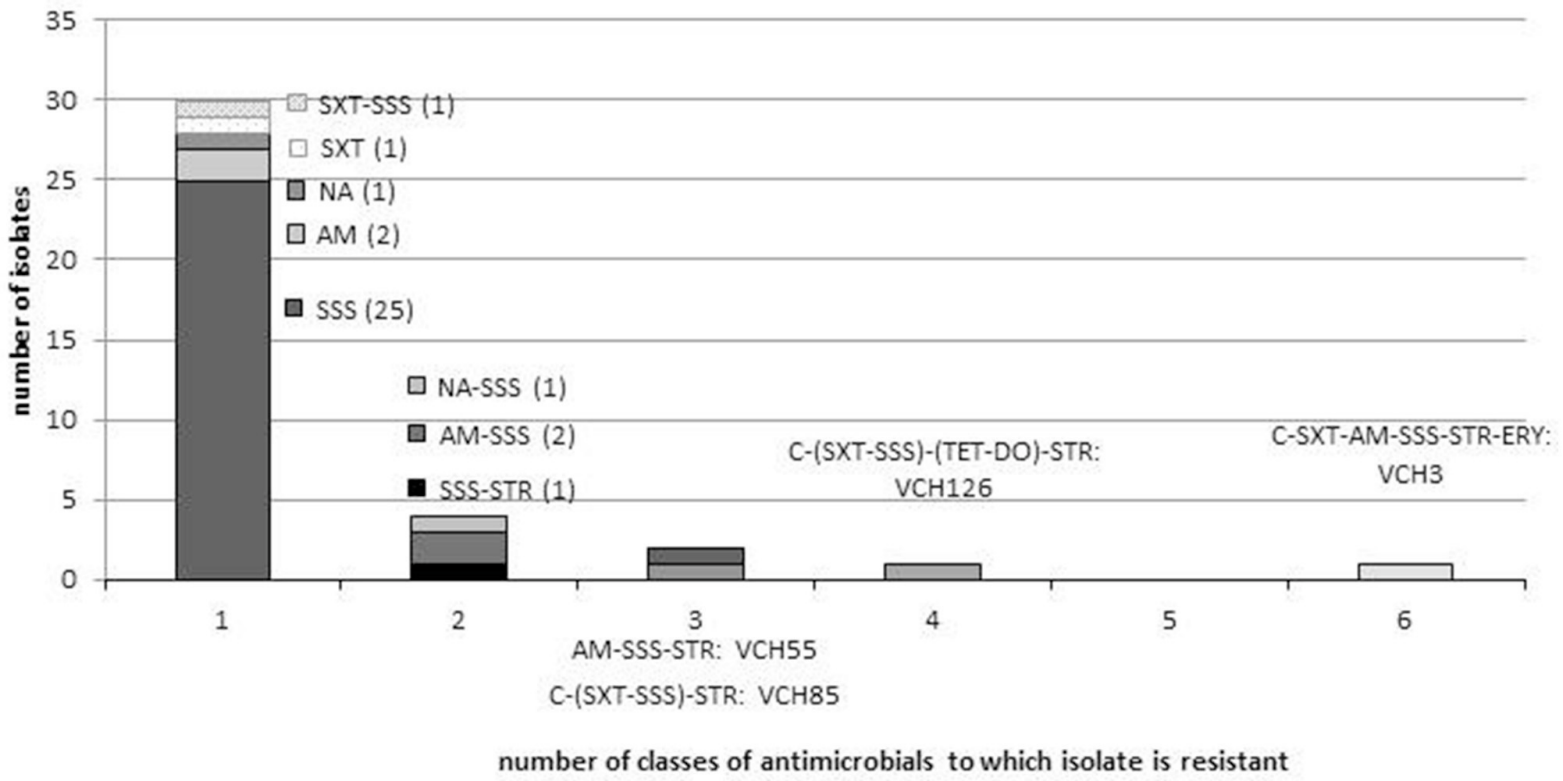
**Distribution of resistance profiles in *V. cholerae* non-O1/non-O139 isolates**. The number of isolates for each resistance profile is indicated in brackets, and isolate identity is indicated only for the four multidrug-resistant strains. AM, Ampicillin; C, chloramphenicol; NA, nalidixic acid; STR, streptomycin; TET, tetracycline; DO, doxycycline; SSS, sulfonamide; SXT, trimethoprim-sulfamethoxazole; ERY, erythromycin. SXT and SSS belong to the class of folate inhibitor pathway and DO and TET to the tetracycline class.

Among the 39 isolates that were resistant to at least one antibiotic, 12 different profiles were observed. The dominant profile was resistance to sulfonamide only (*n* = 26/39; 66.7%), and the 11 other profiles were represented by only one or two isolates (Figure 2). Antimicrobial resistance was not significantly different between LFC and HFC stations (Fisher exact test; *p* = 0.91).

Multidrug resistant isolates are defined as isolates which are resistant to at least three different antimicrobial classes (Magiorakos et al., 2012). Four isolates (10.5%), VCH3, VCH126, VCH85, and VCH55, were multidrug-resistant (Table 4). They displayed four different profiles (Figure 2). The four isolates were all resistant to streptomycin and sulfonamide, but none were resistant to nalidixic acid. Two isolates (VCH3 and VCH126), from stations 2 and 19, were resistant respectively to six and four classes of antimicrobials. They were both resistant to the antimicrobials belonging to the classes of folate pathway inhibitors (trimethoprim-sulfamethoxazole, sulfonamide), phenicols, tetracycline, and aminoglycosides. These phenotypic resistances may be conferred by genes that are frequently associated with the presence of an SXT element. Nevertheless, the class 1 integron integrase gene was detected in one isolate (VCH3) only. VCH3 was also resistant to erythromycin and ampicillin, but susceptible to doxycycline. VCH126 was also resistant to doxycycline.

### Antibiotic resistance genotypes

Seven resistance-associated genes were screened on the 50 isolates, regardless of the resistance profile. We chose to screen for resistance associated with streptomycin (*strA*/*B*) and sulfonamide (*sul1*/*2*), because these genes can be present in the SXT constin, and with erythromycin (*ermA*/*B, mefA*) which is used in Haiti for cholera treatment.

**Figure 3:**
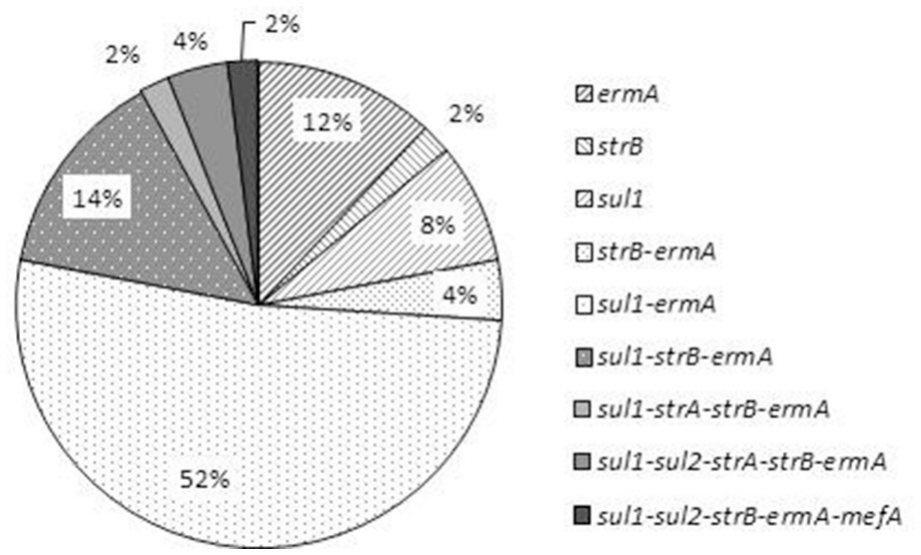
**Distribution of the profiles of resistance-associated genes in the 50 *V. cholerae* non-O1/non-O139 isolates**.

Among the 50 isolates, 78% possessed at least two resistance-associated genes (Figure 3). The genes *sul1, ermA*, and *strB* were detected in the four multidrug-resistant isolates. Three of these isolates harbored five resistance-associated genes and one harbored four genes (Figure 3). The *sul1* gene was detected in 41 (82%) isolates. The *sul2* gene was detected only in three isolates (6.0%) and always in association with *sul1*; these isolates were resistant to sulfonamide. The *strA* gene was detected only in three isolates (6.0%) and always in association with *strB*; these three isolates were resistant to streptomycin. In contrast, the two other isolates that were resistant to streptomycin did not harbor either *strA* or *strB*. *StrB* gene was detected in 11 other isolates that were susceptible to streptomycin. *ErmA* gene was detected in 90.0% of the isolates, while only one strain (VCH3) was resistant to erythromycin; this strain harbored only *ermA*. *Mef* A was detected in only one strain (VCH90) which also carried the *ermA* gene, but was susceptible to erythromycin (Table 4).

## Discussion

Only few studies have investigated the presence of resistance-associated genes in *V. cholerae* non-O1/non-O139 strains in association with phenotypic susceptibility (Raissy et al., 2012; Bier et al., 2015; Bhuyan et al., 2016). One study was carried out on 184 *V. cholerae* non-O1/non-O139 strains of clinical and environmental origin (water and fish), and showed that 11 were resistant to ampicillin, but all were sensitive to the other beta-lactam antibiotics tested, with the exception of four strains resistant to carbapenems (Bier et al., 2015). However, no beta-lactamase-coding genes were found. The other study screened for three resistance-associated genes (*ermB, strA*, and *sul2*) on three strains of *V. cholerae* isolated from seafood in Iran. Only the *strA* gene was detected in one isolate which was resistant to streptomycin, amikacin, and gentamicin (Raissy et al., 2012).

In this study, we detected *strA* in three isolates which were resistant to streptomycin but susceptible to the other aminoglycosides tested. The *ermA* gene was not detected, but the *ermB* gene was detected in 90% of *V. cholerae* isolates, although only one isolate showed a resistance phenotype. This gene, which codes for the methylation of the target, is frequently detected in *Staphylococcus* spp. On the contrary, one strain possessed both the *ermA* gene and the *mefA* gene, which codes for an efflux pump, another mechanism of resistance. Nonetheless, this isolate presented a susceptible phenotype (zone of inhibition = 17 mm).

Our study indicates that the PCR and susceptibility testing approaches are complementary, because they show the presence of genes associated with resistance in susceptible strains and also their absence in resistant strains. This indicates that the link between genes and phenotypes is probably complex. Interestingly, the multidrug-resistant isolates were those that harbored the highest number of resistance-associated genes. Even if a larger panel of associated resistance genes could be investigated, it would not be exhaustive.

Screening for resistance genes is just a first step: it provides information on the reservoir of resistance genes in the autochthonous aquatic *V. cholerae* non-O1/non-O139 population in our collection of isolates. The next step requires the localization of these genes (chromosome, plasmid, ICE) to estimate their potential for dissemination more precisely. Some of these resistance-associated genes are associated with mobile genetic elements. The s*ul1*/*2* genes have been detected in class 1 integrons or/and in plasmids in *V. cholerae* O1 (Dalsgaard et al., 2001; Iwanaga et al., 2004; Ceccarelli et al., 2006) and in non-O1/non-O139 serogroups (Dalsgaard et al., 2000). The *strA*/*B* genes are associated with the class 1 integron as well as the SXT element (Hochhut et al., 2001; Iwanaga et al., 2004) in *V. cholerae* O1. In this study, a class 1 integron was detected in only one strain (VCH3), which showed the expected multidrug resistance (chloramphenicol, trimethoprim-sulfamethoxazole, ampicillin, tetracycline, and sulfonamide) that can be harbored by the SXT/R391 integrative and conjugative element (ICE). In Haiti, Ceccarelli et al. (2013) described the presence of two types of SXT/R391 ICEs displaying different genetic organizations, one in O1 serogroup strains (ICEVchHai1) and another (ICEVchHai2) in some clinical isolates of non-O1/non-O139 serogroups (Ceccarelli et al., 2013). ICEVchHai2 lacks the antibiotic resistance cluster typically inserted in variable region 3. It remains to be seen whether VCH3 harbors an ICE and, if so, which genetic organization it possesses.

Considering the Haitian situation, we compared the susceptibility profiles of the non-O1/non-O139 isolates with those of *V. cholerae* O1 of either clinical or environmental origin, which have been published since the beginning of the cholera outbreak in October 2010 (Sjölund-Karlsson et al., 2011; Talkington et al., 2011; Alam et al., 2014, 2015; Folster et al., 2014). Our results clearly indicate that the non-O1/non-O139 *V. cholerae* isolated from surface waters showed phenotypical antimicrobial patterns different from those of the epidemic strain.

Two collections of toxigenic *V. cholerae* O1 strains have been tested for antimicrobial susceptibility: 122 laboratory-confirmed *V. cholerae* O1 clinical isolates, recovered by the National Public Health Laboratory in Haiti from October 2010 to January 2011 (Sjölund-Karlsson et al., 2011) and 17 toxigenic *V. cholerae* O1 isolates collected from surface waters in the West Department from April 2013 to March 2014 (Alam et al., 2015). These *V. cholerae* O1 strains, regardless of their origin (clinical or environmental) all displayed the same multidrug resistance profile: resistance to streptomycin, sulfamethoxazole, trimethoprim-sulfamethoxazole, and nalidixic acid. The 50 *V. cholerae* non-O1/non-O139 strains collected in our study displayed 12 different profiles of resistance but only 8.0% of them were multidrug resistant. Two multidrug-resistant strains isolated from wastewater were resistant to four and six families of antibiotics. None of the four multidrug-resistant non-O1/non-O139 isolates studied displayed the phenotypical profile of the O1 serogroup. In the Alam et al. (2015) study, all 17 isolates of *V. cholerae* O1 from aquatic environments were susceptible to doxycycline, tetracycline, chloramphenicol, and ampicillin. In contrast, among the non-O1/non-O139 isolates in this study, some were resistant to doxycycline (1 strain), tetracycline (2 strains), chloramphenicol (3 strains), and ampicillin (6 strains), and one was resistant to erythromycin.

In another collection of 1029 *V. cholerae* O1 strains collected from 18 towns in Haiti from April 2012 to March 2013, the 115 *V. cholerae* tested by CDC Atlanta (Steenland et al., 2013) showed 100% susceptibility to ampicillin and to tetracycline, whereas a fraction of our *V. cholerae* non-O1/non-O139 were resistant to ampicillin and tetracycline (respectively 12.0 and 4.0%).

These differences of susceptibility profile between the strains of *V. cholerae* non-O1/non-O139 studied and the profile of O1 epidemic strains of Haiti could be partly linked to a difference in the history of exposure to antibiotics. Given that *V. cholerae* non-O1/non-O139 serogroups isolates studied were collected in aquatic environment, we could rise the hypothesis that they have not experienced the same selection pressure as the toxigenic *V. cholerae*. Therefore, because doxycycline, tetracycline, and erythromycin are currently used in the treatment of diarrheal diseases in Haiti, the resistance acquired here by aquatic *V. cholerae* non-O1/non-O139 to these antibiotics may be due to selection pressure on local enteropathogenic bacteria. Accordingly, among the four multidrug-resistant isolates of *V. cholerae* non-O1/non-O139 detected in this study, the two strains harboring resistance to the most antibiotics (four and six different classes of antibiotics, respectively, for VCH126 and VCH3) came from raw wastewater (VCH 126 in Gonaives and VCH3 in Martissant) and these two strains were resistant to two of the three antibiotics used locally: VCH126 was resistant to tetracycline and to doxycycline and VCH3 was resistant to erythromycin and tetracycline but not to doxycycline.

In a previous study, Katz et al. discovered that the epidemic clone is poorly transformable by horizontal gene transfer, and they found no evidence that environmental strains have played any role in its evolution (Katz et al., 2013). This could explain that the susceptibility profile of the epidemic strain has not changed since the beginning of the outbreak, except the single observation of the variant 2012EL-2176 isolated from a clinical case in 2012. That single variant showed the typical resistance phenotype of the outbreak strain, but additional resistance to ampicillin had been acquired and the minimum inhibitory concentration of tetracycline had become intermediate (Folster et al., 2014). Resistance to ampicillin and tetracycline were both found in our non-O1/non-O139 isolates.

Nevertheless, to have information about the possibility of genetic exchanges between non-O1/non-O139 *V. cholerae* isolates and the O1 epidemic clone, deeper genetic investigations are necessary for example by whole genome sequencing, determination of the MLST profile or comparison of mutations on targeted genes (*ctxB, QRDR*, etc.).

The prevalence of antibiotic resistance in non-O1/non-O139 *V. cholerae* in an endemic zone of cholera has also been studied in Sanitpur, Assam, North East India (Bhuyan et al., 2016). A collection of 107 strains of *V. cholerae* (among which a single strain of O1 Ogawa) was isolated from 38 water samples (river Brahmapoutra and its tributaries, canal, tea garden) before the rainy season and during monsoon (flooding) in 2012 and 2014. Antibiotic susceptibility was tested by the same disk diffusion method, allowing comparisons with our results. The dominant resistance to sulfonamide in Haïti (70%) was consistent with the dominant resistance to sulfamethoxazole (around 60%). Resistance to two antibiotics was much more frequent in Assam than in Haïti: streptomycin (respectively around 50 *vs*. 10%), and nalidixic acid (around 50 *vs*. 4%). Resistance to three antibiotics was slightly more frequent in Assam than in Haïti: Ampicillin (around 35 *vs*. 12%), Erythromycin (around 15 *vs*. 2%), and tetracycline (around 10 vs. 4%). Two antibiotics gave opposite results: resistance to chloramphenicol was present in Haiti (6%) and absent in Assam; on the contrary, resistance to ciprofloxacin was present in Assam (5%) and absent in Haïti. These differences may be linked to sampling fluctuations but could also be linked to different choices in the two countries for diarrheal diseases and severe cholera treatment.

Thus, in cholera endemic context, the presence of *V. cholerae* non-O1/non-O139 with reduced susceptibility or resistance to antibiotics used for human medicine in the aquatic environment may constitute a mild public health threat. Although the risk of therapeutic failure for infections by a few multidrug-resistant strains seems limited (and the incidence of such infections is still unknown in Haiti), the screening for non-O1/non-O139 serogroups should be included in surveillance programs on diarrhea outbreaks. Moreover, the hypothesis that the aquatic population of *V. cholerae* may constitute a reservoir of resistance genes, supplied by genetic exchanges *in situ* with enteric bacteria such as *E. coli*, which do not survive in aquatic environments, still needs to be investigated.

## Conclusion

In the context of a cholera outbreak in Haiti, non-O1/non-O139 *V. cholerae* in surface waters showed antimicrobial patterns different from the epidemic strain: there were few multidrug-resistant strains and a high diversity of resistance profiles, including resistance to doxycycline, tetracycline and erythromycin. In contrast, according to the literature, all the clinical and environmental isolates of toxigenic serogroup O1 in Haiti were susceptible to these three antibiotics or had reduced susceptibility (tetracycline) and all presented the same specific multidrug-resistance profile.

However, the presence of acquired resistance to antibiotics among the autochthonous *V. cholerae* non-O1/non-O139 in the aquatic environment can be construed as the result of a local selection pressure on enteric bacteria in Haiti, where the use of antibiotics is not strictly regulated.

Further research is required to address this public health issue. The ability of the autochthonous aquatic population of *V. cholerae* non-O1/non-O139 to acquire and transfer resistance genes, especially in wastewaters, needs to be investigated. In addition, the contribution of the non-O1/non-O139 serogroups to co-infections of cholera cases, or to diarrhea cases in Haiti, should be documented as part of national surveillance programs of gastro-intestinal infections.

## Author contributions

SB, contributed to the design of the work, performed the field study, contributed the acquisition, the analysis and the interpretation of the data, participated to the assays, and wrote the paper. JL, contributed to the design of the work, performed the field study and the interpretation of the data, participated to the assays, and wrote the paper. EL, contributed the acquisition, the analysis and the interpretation of the data for the work, participated to the assays, and revised the paper. RP, contributed to the design of the work, revising the work and final approval of the version to be published. SR, contributed to the design of the work, revising the work and final approval of the version to be published. EJ, contributed to the analysis and the interpretation of the data, and revised the paper. IK, contributed to the analysis and the interpretation of the data, and revised the paper. JB contributed to the acquisition of the data and revised the manuscript.

## Funding

The field study was funded by a grant from the French Embassy in Haiti supporting trans-national cooperation between the French Agency for Food Environmental and Occupational Health and Safety (ANSES) and the National Laboratory of Public Health in Haiti.

### Conflict of interest statement

The authors declare that the research was conducted in the absence of any commercial or financial relationships that could be construed as a potential conflict of interest.
